# Regulation of a Vascular Plexus by *gata4* Is Mediated in Zebrafish through the Chemokine *sdf1a*

**DOI:** 10.1371/journal.pone.0046844

**Published:** 2012-10-03

**Authors:** Ingrid Torregroza, Audrey Holtzinger, Karen Mendelson, Ting-Chun Liu, Timothy Hla, Todd Evans

**Affiliations:** 1 Department of Surgery, Weill Cornell Medical College, New York, New York, United States of America; 2 Department of Pathology and Laboratory Medicine, Weill Cornell Medical College, New York, New York, United States of America; Instituto de Medicina Molecular, Portugal

## Abstract

Using the zebrafish model we describe a previously unrecognized requirement for the transcription factor *gata4* controlling embryonic angiogenesis. The development of a vascular plexus in the embryonic tail, the caudal hematopoietic tissue (CHT), fails in embryos depleted of *gata4*. Rather than forming a normal vascular plexus, the CHT of *gata4* morphants remains fused, and cells in the CHT express high levels of osteogenic markers *ssp1* and *runx1*. Definitive progenitors emerge from the hemogenic aortic endothelium, but fail to colonize the poorly vascularized CHT. We also found abnormal patterns and levels for the chemokine *sdf1a* in *gata4* morphants, which was found to be functionally relevant, since the embryos also show defects in development of the lateral line, a mechano-sensory organ system highly dependent on a gradient of *sdf1a* levels. Reduction of *sdf1a* levels was sufficient to rescue lateral line development, circulation, and CHT morphology. The result was surprising since neither *gata4* nor *sdf1a* is obviously expressed in the CHT. Therefore, we generated transgenic fish that conditionally express a dominant-negative *gata4* isoform, and determined that *gata4* function is required during gastrulation, when it is co-expressed with *sdf1a* in lateral mesoderm. Our study shows that the *gata4* gene regulates *sdf1a* levels during early embryogenesis, which impacts embryonic patterning and subsequently the development of the caudal vascular plexus.

## Introduction

A characteristic feature of organ morphogenesis is the coordinated cell migration of groups of cells, resulting in the appropriate organ position, shape and size. The *gata4* gene is known to play key roles in the development and maintenance of several organ systems, including those comprising cardiovascular, reproductive, and digestive tissues [1], [2]. The gross phenotype of the mouse *Gata4* mutant is defective embryonic folding, caused indirectly due to unknown alterations in extra-embryonic endoderm [3], [4]. Conditional mouse mutants [5], [6], [7] and studies in zebrafish [8] defined functions for *gata4* in early embryonic organ formation, including heart and liver. The mutants display morphogenetic defects, rather than a failure in cell specification or differentiation, because those functions are compensated by the sister genes *gata5* and *gata6* [9], [10]. The morphogenetic genes that *gata4* regulates remain largely unknown, although they include cell cycle regulators [11] and signaling molecules including BMPs and WNT inhibitors [12], which can function by non-cell-autonomous mechanisms. Given the pleiotropic nature of the *gata4* mutant phenotype, the question arises whether a common morphogenetic program controlled by *gata4* functions in different organ systems, or if varied tissue-specific programs are downstream of *gata4* to control development of distinct organ systems.

We developed a loss-of-function model for *gata4* in the zebrafish system using anti-sense morpholinos to block expression of the gene during embryogenesis [8]. An advantage of the zebrafish model is that it lacks the requirement of *gata4* in extra-embryonic tissues, which in the knockout mouse leads to early embryonic lethality. Zebrafish embryos depleted of *gata4* are defective for normal heart tube growth and looping, with a phenotype remarkably similar to mouse embryos lacking *Gata4* in the embryo proper, and rescued for extra-embryonic function by tetraploid complementation [8], [13]. In contrast to mouse mutants, the morphant fish embryos continue to survive even in the absence of a normal heart, and this allowed us to identify an additional function for *gata4* in gut-derived organ growth. The finding that *gata4* is needed for growth of both heart and gut-derived organs is not unexpected, since the gene is expressed in progenitors that contribute to these organ systems from early stages of embryogenesis. Here we revisit the phenotype of *gata4* morphant embryos, and describe for the first time a function for *gata4* in the development of the vascular system, specifically the caudal hematopoietic tissue (CHT). We show that *gata4* regulates angiogenesis that normally forms this plexus during a “fetal-like” intermediate stage of hematopoiesis. This observation was unexpected because *gata4* is not obviously expressed in the hematopoietic progenitors or the CHT.

Considering that the function of *gata4* might be indirect, we discovered another previously uncharacterized phenotype, occurring in the lateral line of *gata4* morphants. The lateral line is an ectodermal placode-derived system comprised of a set of pressure-sensitive sensory organs, the neuromasts, distributed in a stereotypic pattern along the body surface [14]. A major determinant of the collective cell migration required for lateral line development is the chemokine stromal-derived factor 1 or *sdf1a* (also called *cxcl12a*), mediated by *cxcr4*/*cxcr7* chemokine receptor signaling [15], [16], [17], [18]. This ligand is well characterized as a chemo-attractant controlling the migration of neurons and primordial germ cells, lymphocyte trafficking, and hematopoietic stem cell (HSC) homing [19], [20], [21]. In the lateral line, the pathway coordinates the migration and timely deposition of collective groups of cells that form neuromasts. Therefore, chemokine signaling also regulates tissue migration and adherence in order to facilitate organ system development. We find that the novel functions for *gata4* in angiogenesis and sensory organ development can both be ascribed to deregulated expression of the *sdf1a* gene.

## Methods

### Ethics Statement

The only animals used were zebrafish. All experiments using zebrafish were previously approved following full review of the Weill Cornell Medical College Institutional Animal Care and Use Committee (IACUC), on file as protocol 2011-0100, which expires February 2015. Weill Cornell Medical College operates its NIH approved Animal Care and Use program under the Animal Welfare Assurance number A3290-01.

### Zebrafish Strains

Zebrafish were raised and staged according to Westerfield [22]. The *tg(gata1:dsRed)* line [23] was provided by Shou Lin (UCLA, Los Angeles, CA). The *tg(fli1:egfp)* [24] was purchased from the Zebrafish International Resource Center (Eugene, OR). The *tg(claudinb:gfp)* line [17] was provided by James Hudspeth (Rockefeller University, New York, NY). The *tg(cd41:gfp)* strain [25] was provided by Robert Handin (Brigham and Women’s Hospital, Boston, MA). The dominant-negative isoform for *gata4* was generated essentially as described [26], but using the zebrafish *gata4* cDNA clone for isolation of the DNA-binding domain, cloned in frame with the SR domain from mouse. A region of the zebrafish *gata4* cDNA encompassing both zinc fingers and the entire DNA-binding domain (from amino acids 131–338) was isolated by PCR using primers that incorporated a stop codon and permitted in-frame fusion at the N-terminus with sequences encoding the strong repression domain (SR; amino acids 1–69) of the mouse Mxi1 gene, or a valine-proline mutation of the SR domain, SR-P [27]. PCR primers for *gata4* were F: (TE2477), GAAGATCTTGGACGGCTTCGCAC.

R: (TE2476), AACTCGAGTTAGGATCCGCTTGGAGA.

The chimeric cDNA encoding a protein called SR-Gata4 was confirmed by sequencing. The chimeric cDNA was cloned into the middle entry vector in the Gateway Cloning Kit using the In-Fusion™ Advantage PCR kit (Clontech) and primers:

F: (TE2542), TATAGGGCGAATTGGGTACCATGGAGCGGGTGCGGATGATCAA,

R: (TE2543), GGGAACAAAAGCTGGAGCTCTTAGGATCCGCTTGGAGAGCCCATA and recombined with the hsp70I promoter vector and IRES driving RFP vector into the tol2 destination vector. DNA was co-injected with RNA encoding Tol2 recombinase, and potential F0 founders were tested for transmission of the transgene, and inducible expression of the *sr-gata4* RNA. Several independent lines were isolated and maintained as *tg(hsp70:sr-gata4)*. Characterization of SR and SR-P constructs was performed as described [26]. For gel mobility-shift experiments, the probe (top strand) was 5′-TCCATCTGATAAGACTTATCTGCTGCC. The same (unlabeled) sequence was used as specific competitor, while the control mutant sequence (top strand) was: 5′-TCCATCTCTTAAGACTTAAGTGCTGCC.

### Morpholino Microinjection

All morpholinos were purchased from Gene-Tools, LLC. The sequence of the *gata4* translation blocker is (5′-TCCACAGGTGAGCGATTATTGCTCC-3′). It was injected in concentrations ranging from 10–12 ng, which is sufficient to reproducibly generate the full range of morphant phenotypes in essentially 100% of the embryos. The morpholino targeting *sdf1a* is also a translation blocker, validated in multiple previous studies [16], [17], [18] and shown to phenocopy an *sdf1a* mutant; its sequence is 5′- TTGAGATCCATGTTTGCAGTGTGAA-3′. For rescue experiments, embryos were injected first with the *gata4*-specific morpholino, and a cohort of these morphants injected subsequently with the *sdf1a*-specific morpholino at concentrations between 0.25–4ng, which is below the threshold for generating an *sdf1a* morphant phenotype.

### Whole Mount in-situ Hybridization and Di-2-ASP Staining

Whole mount *in situ* hybridization was performed as described [28]. Briefly, embryos were fixed in 4% paraformaldehyde at defined stages. Embryos were treated with 10 µg/ml proteinase K at 22C for 8 min (30 hpf), 10 min (36 hpf), or 20 min (48 hpf). Hybridization was performed at 70°C, in 57%–65% formamide buffer with digoxigenin-labeled anti-sense RNA probes. The *l-plastin* [29] and *runx1* [30] probes were as described. Probes for *sdf1a* and *spp1* were generated from full-length cDNA clones purchased from Open Biosystems.

To stain the lateral line neuromasts, 3–5 day old embryos were cultured in system water containing 5% BSA and 200 uM 4-[4-(diethylamino)styryl]-N-methylpyridinium iodide (4-Di-2-ASP, Invitrogen) for 5 minutes. They were then washed in system water, and observed under fluorescence using a GFP filter.

### Microscopy

All *in situ* hybridization and 4-Di-2-Asp images were taken with a Nikon SMZ 1500 microscope using a Spot Insight Firewire digital camera and advanced Spot imaging software (Morell Instruments Company, NY). All other images were acquired with a Zeiss Observer.Z1 microscope and a Zeiss Axiocam digital camera (Carl Zeiss MicroImaging). The Axiovision40 V4.8.1 software was used to collect Z-stacks of the images which were then flattened by using the extended focus feature. For confocal analysis, fixed embryos were mounted in 1% low melt agarose (National Diagnostics) dissolved in water. Confocal images were taken with an Olympus Fluoview Microscope with a 60× lens and analyzed using Fluoview software. Images were processed with ImageJ (1.42 q), Imaris (Bitplane Inc.), and Adobe PhotoshopCS4 software.

### Quantitative PCR

Total RNA was isolated from embryos using the RNeasy kit (Qiagen). First-strand cDNA synthesis was performed using the Superscript III kit (Invitrogen). The cDNA was analyzed with the Light Cycler 480 II SYBR green master mix (Roche), and using the Light Cycler 480 II machine (Roche). All samples were prepared in triplicate, and each experiment was repeated at least 3 times using independent batches of embryos. The PCR cycle conditions were 95°C for 5 minutes, (95°C for 10 seconds, 54°C for 10 seconds, and 72°C for 15 seconds) for 40 cycles. The data were analyzed to determine Cp values using the 2^ΔΔT^ method [31], normalized to transcript levels for β-actin. Relative morphant transcript levels were quantified considering wild-type levels as 1. Primers were:

*ae3globin*, For: 5′-GACCTAAGCCCCAACTCTCC,

Rev: 5′-GTCAGCAAACCTCCCTTCAG

*runx1*, For: 5′- AGAGCTGACGGCGTTCAC, Rev: 5′- CATGGCTGACATGCCAATAC

*lmo2*, For: 5′- TGGGACGCAGGCTTTACTAC, Rev: 5′- AGTCCGTCCTGACCAAACAG

*cmyb*, For: 5′- TGATGCTTCCCAACACAGAG, Rev: 5′- AGGTATTTGTGCGTGGCTTC

*sdf1a*, For: 5′- ATTCGCGAGCTCAAGTTCC, Rev:5′- TGCACACCTCCTTGTTGTTC

*bactin*: For: 5′-GACAACGGCTCCGGTATG, Rev:5′-CATGCCAACCATCACTCC

## Results

### Gata4 Regulates Vascular Development

**Figure 1:**
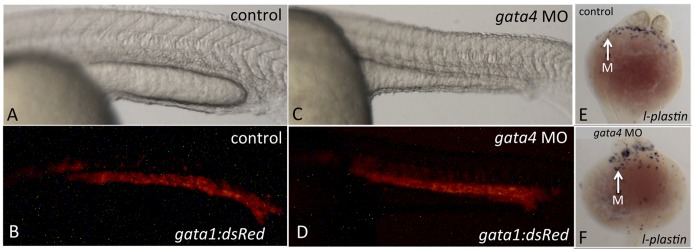
Hematopoiesis initiates normally in the gata4 morphant. Control (A, B) and *gata4* morphant (C,D) embryos derived from *the tg(gata1:dsred)* reporter fish show that primitive erythropoiesis in the ICM is normal in *gata4* morphants. A and C are brightfield; B and D are the fluorescent views. Views are lateral, anterior to the left. Control (E) and *gata4* morphant (F) embryos stained for the macrophage marker *l-plastin* show that primitive myeloid development is also normal in the *gata4* morphants. White arrows indicate blue-stained macrophages (M). Views are ventral, anterior to the top. For each, n >50.

**Figure 2:**
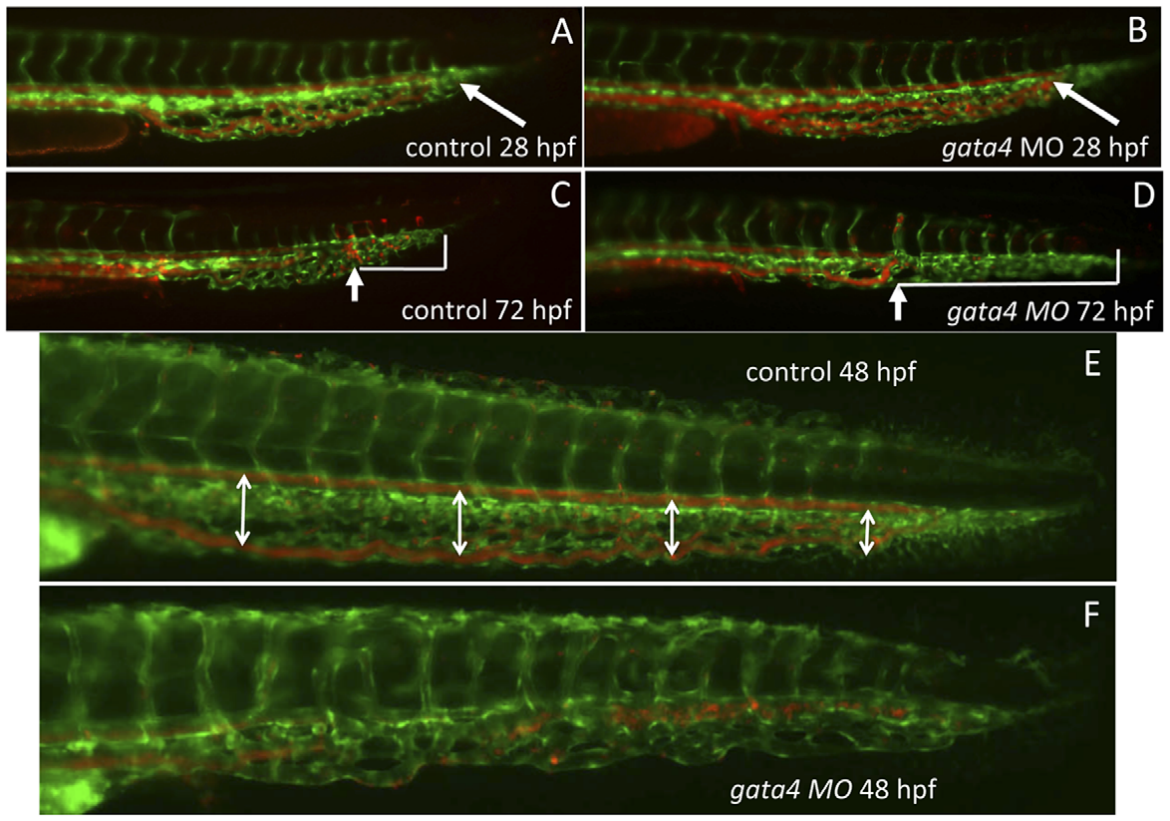
The vascular morphology of the CHT is disrupted in the *gata4* morphant embryo. Shown are representative embryos (n >50) derived from *tg(gata1:dsred; fli1:egfp*) double transgenic reporter fish. The green signal shows the vascular endothelium, while the red signal shows the circulation of blood cells, which are moving so quickly that individual cells are not seen. At 28 hpf (A,B) the blood circulates to the caudal end of the developing CHT (the caudal end is indicated by white arrows) in both control uninjected (A) and *gata4* morphant (B) embryos. However, by 72 hpf (C,D), the vascular plexus is much more developed in the control (C) compared to the morphant (D). In addition, the blood circulation is restricted in the morphant, failing to move into the more caudal regions, as indicated in panels C and D by the white brackets that mark the distance from the caudal end of the CHT to the most caudal position of circulating cells (indicated by the white arrows). The block to plexus formation can be seen clearly at 48 hpf (E,F), when in the control embryos (E) the dorsal aorta and caudal vein are well separated to create a fenestrated space (this space is indicated by double-headed arrows), which is not seen in the morphant (F). MO = morphant. Views are lateral, anterior to the left.

In a previous study [8], we noted that by 4 days post fertilization (dpf) there is a marked absence of blood flow through the improperly looped but still beating heart of the *gata4* morphant embryo. This was unexpected since *gata4* is not thought to be expressed in the hematopoietic system. Furthermore, although the morphant heart is abnormal, it continues to beat, and the embryonic morphant circulatory system is at least grossly normal. Therefore, we investigated the basis for the hematopoietic deficit. The initiation of hematopoiesis appears normal in *gata4* morphants based on *in situ* hybridization experiments to detect transcripts for the erythroid transcription factor *gata1* (not shown), and by imaging RFP+ cells in *tg(gata1:dsRed)* reporter fish (Fig. 1A–D). Also, *in situ* hybridization experiments using an *l-plastin* probe showed that early myelopoiesis is also not affected by loss of *gata4* (Fig. 1E,F). Therefore, the initial generation of “primitive” waves of hematopoiesis is not regulated by *gata4*. Although the embryonic vasculature including angiogenesis of the intersomitic vessels (ISVs) is grossly intact (see Fig. 2), the heart defect could lead to a loss of blood flow and pooling of the blood at later stages. We used quantitative flow cytometry experiments to compare the number of erythroid cells in control and *gata4* morphant embryos derived from *tg(gata1:dsRed)* reporter fish. In morphants, the number of RFP+ cells is significantly decreased by 4 dpf to a level on average of 53% the number in control embryos (n = 14 independent batches, p<0.001). The embryos live for several more days, but hematopoiesis does not recover. As in the mouse, several “waves” of definitive hematopoietic progenitors have been described during early zebrafish embryogenesis [32]. Between 30–36 hpf, erythro-myeloid committed progenitors (EMPs) arise in the posterior blood island, derived from *lmo2*+ lateral mesoderm [33]. Around this same time, cells that are thought to be the long-term adult HSCs begin to emerge from the floor of the dorsal aorta and subsequently migrate to the same region of the posterior tail, which has matured into a more vascularized niche, termed the CHT [34]. These aorta-derived HSCs do not contribute to erythropoiesis until a later stage compared to the time we document defects in hematopoiesis for the *gata4* morphant (4 dpf). This suggests that the *gata4* morphants have a late defect in primitive erythropoiesis, correlating with an intermediate stage during which time the CHT plexus develops.

**Figure 3:**
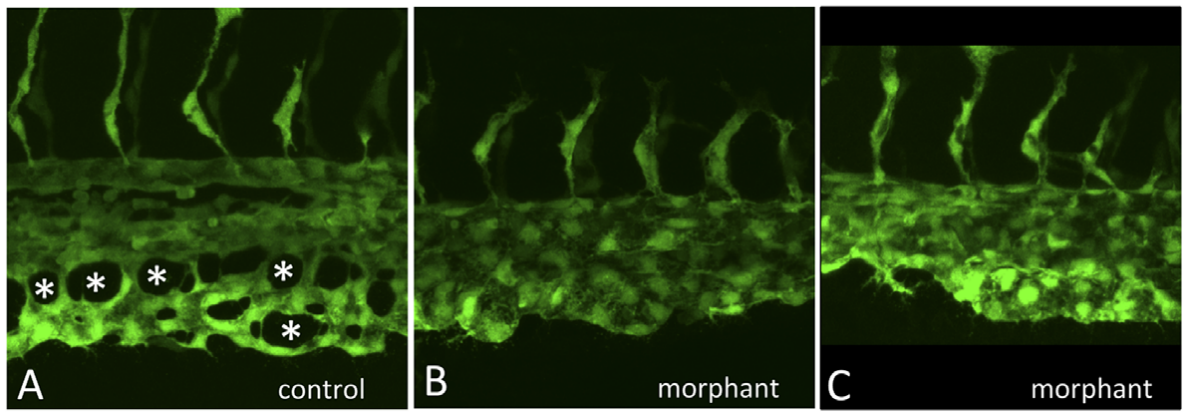
The gata4 morphant CHT fails to develop fenestrated vascular structures. Shown are higher resolution images of control (A) and two independent examples of morphant embryos (B,C) imaged at 32 hpf by confocal microscopy, as the caudal plexus is forming. While vasculogenesis initiates normally, and intersegmental vessels sprout equivalently, angiogenesis in the CHT of control embryos generates a vascular plexus containing open fenestrated structures, indicated by the asterisks in A, that are missing in *gata4* morphants.

To investigate this intermediate stage, the development of the CHT was evaluated in *gata4* morphants using embryos derived from double transgenic *tg(fli1:gfp; gata1:dsred)* fish, to follow the co-development of CHT vasculature and circulating erythroid cells [34]. As shown in Fig. 2 (panels A,B) the CHT of *gata4* morphants appears normal at 28 hpf, and erythroid cells circulate through the tail as in controls. However, by 3 dpf there is a significant disruption in the morphology of the CHT in *gata4* morphants compared to controls (Fig. 2C,D). Circulation does not extend deeply into the tail and is limited to a much more proximal region of the trunk (note the length of the blue bracket indicating the morphant tail that lacks circulation in Fig. 2D). The vascular plexus is significantly reduced in size and extent of branching. The phenotype can be seen already by 48 hpf, when a clearly defined plexus between the dorsal aorta and caudal vein is seen in control embryos (Fig. 2E, denoted by the white arrows), which is not seen in the morphant embryos (Fig. 2F). Confocal microscopy was used to evaluate with higher resolution angiogenesis associated with the CHT. As shown in Fig. 3, the normal CHT plexus develops with a series of looped structures (indicated by asterisks in Fig. 3A). These structures are missing in the *gata4* morphants, which develop a smooth unfenestrated tissue lacking looped structures (Fig. 3, panels B,C). Thus, while the primitive hematopoietic and vasculogenic programs initiate normally, *gata4* morphants are disturbed subsequently for development and vascularization of the CHT, and this correlates temporally with a relative loss of circulating blood cells by 4 dpf. The failure to form a normal CHT plexus is not simply an indirect defect caused by a poorly functioning heart in the *gata4* morphant. Two independent morphants that also display similar cardiac morphogenetic phenotypes and poor circulation (*gata5* and *tbx2a*) have normal CHT plexus formation (Supplemental Fig. S1). Previously, Choi et al. demonstrated using defined mutants (*tnnt2* and *smo*) that circulation is not required for the formation of the plexus in the CHT, although it can affect the subsequent stability of the structure [35].

**Figure 4:**
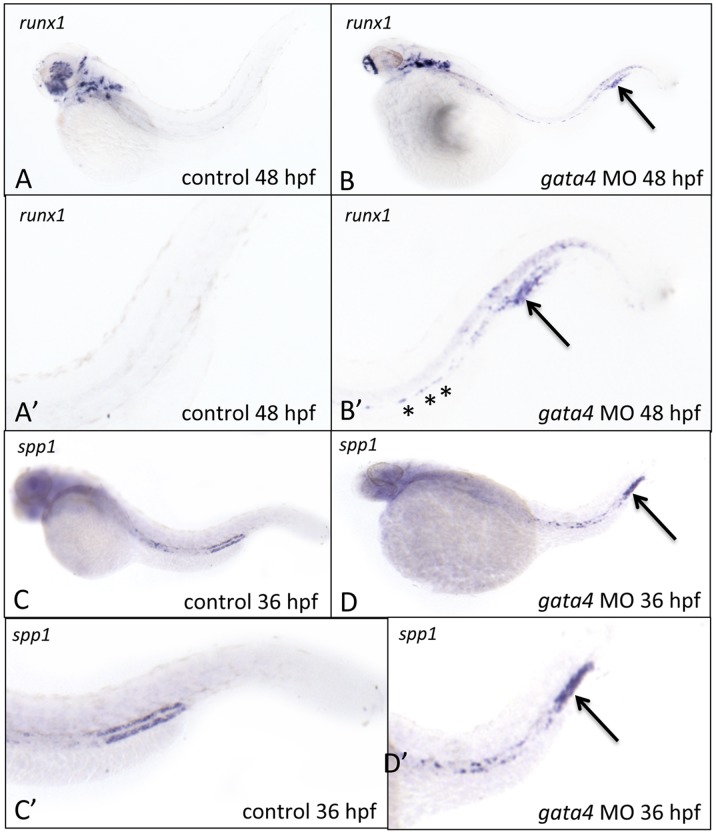
The CHT in *gata4* morphants shows aberrant expression of runx1 and osteopontin. A–D: Shown are representative control (left panels) or *gata4* morphant embryos following *in situ* hybridization to detect expression of *runx1* at 48 hpf (A–B) or *spp1* at 36 hpf (C–D). A’–D’ are higher magnification views of the tails from panels A–D, respectively. Asterisks in B’ indicate the presence of remaining *runx1*+ cells associated with the dorsal aorta in morphants. The strong expression in the tails of *runx1* and *spp1*, indicated by the arrows, was reproducibly noted in 60% and 50% of *gata4* morphants, respectively (n = 20 each per experiment). Views are lateral, anterior to the left.

HSCs are derived from hemogenic endothelium at the floor of the dorsal aorta [36], [37], and can be phenotypically identified by expression of HSC markers including *cd41* [38] and *runx1* [39]. To further evaluate circulation into the CHT, we analyzed the development of progenitors in morphant embryos from the *cd41:gfp* transgenic reporter line and confirmed that GFP+ cells emerge from the dorsal aorta similar to wildtype embryos. However, unlike control embryos, the GFP+ cells are never detected subsequently associated with the CHT (Supplemental Fig. S2). By 48 hpf, *runx1+* progenitors in control embryos are found in the CHT or in circulation. However, at this same stage, *runx1*+ cells remain scattered along the midline of morphant embryos (Fig. 4A,B; see asterisks in panel B’). In addition, a strong patch of *runx1* staining is found at 48 hpf associated with the CHT, which is never seen in control embryos (Fig. 4B, arrow). In addition to its function as a cell-autonomous regulator of HSC [30], [39], *runx1* is expressed in prechondrocytic tissue that forms embryonic cartilage, and in periosteal and perichondral membranes of bone [40], [41]. We evaluated the expression pattern for various known bone marrow stromal factors and found a striking accumulation of transcripts for osteopontin (*spp1*), also associated with this *runx1*+ domain of the morphant CHT at 48 hpf (Fig. 4C,D). Therefore, when *gata4* is depleted, definitive progenitors are born in the hemogenic endothelium. We do not know the fate of these progenitors, but based on the abnormal vascularization of the CHT, it appears that they fail to seed the CHT. At the same time, the CHT region expresses aberrantly at least some markers normally associated with osteochondrogenic tissue.

### The *sdf1a* Expression Pattern is Altered in *gata4* Morphants

**Figure 5:**
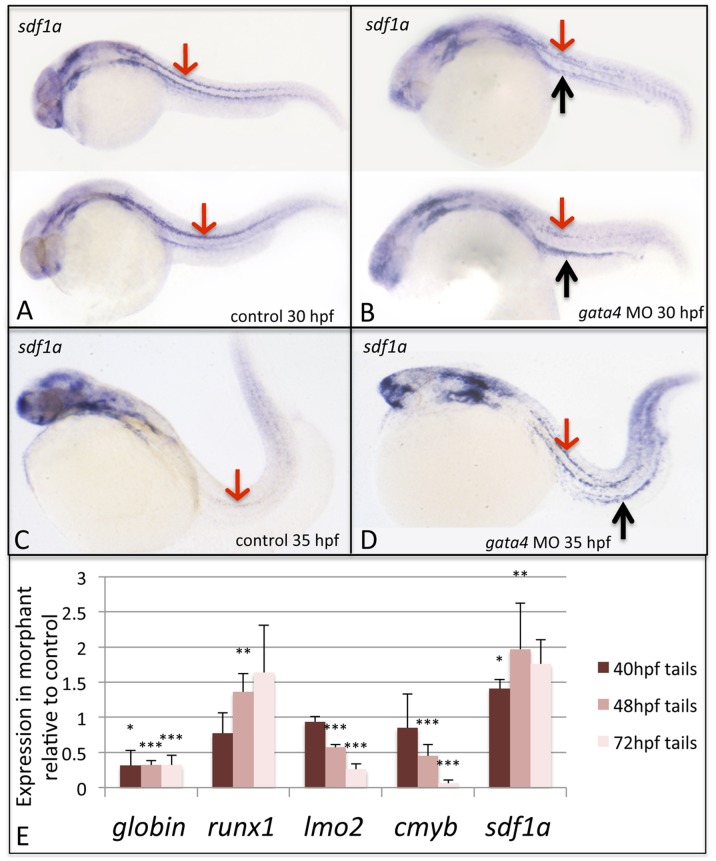
The *sdf1a* expression pattern is disturbed in *gata4* morphants, while quantitative analysis confirms changes in expression in morphant embryos for *sdf1a* and hemato-vascular markers. Shown are representative embryos (n >50) following *in situ* hybridization to detect expression of *sdf1a* transcripts in control (A,C) and morphant (B,D) embryos, at 30 hpf (A,B) and 35 hpf (C,D). In panels A and B, 2 independent embryos are shown representing the range of patterns detected. Red arrows pointing down show the strong stripes of expression in lateral mesoderm in control embryos, which is nearly gone by 35 hpf. In contrast, the pattern is less clear in mesoderm of 30 hpf morphant embryos, but strongly persists by 35 hpf, and is much enhanced in the region associated with the pronephric tubules, particularly at 35 hpf (black arrows pointing up in panels B and D). Views are lateral, anterior to the left. (E) Tails including the CHT region were isolated from control and *gata4* morphant embryos at 40, 48, or 72 hpf and processed by qPCR to measure relative levels of ae3*globin*, *runx1*, *lmo2*, *cmyb*, and *sdf1a*. The Y axis plots the relative transcript levels of morphants compared to controls (controls set at a value of 1). Each sample included >30 embryos, and data was collected in 3 independent experiments. Student’s T-test was used to determine significance (*p<0.05, **p<0.01, ***p<0.001).

In addition to *spp1*, the expression patterns for additional known stromal factors were evaluated in the *gata4* morphants, including *ang1*, *jagged1*, *ncad*, *sdf1a, and sdf1b*. The expression patterns for ang1, jagged1, ncad, and sdf1b appeared unchanged in the gata4 morphant (data not shown). Only *spp1* showed the aberrant pattern in the CHT, but an abnormal expression pattern was also found for the chemokine *sdf1a* (Fig. 5). Transcripts for *sdf1a* are normally found between 24–48 hpf at defined structures of the head, pronephric duct, and in a single stripe of mesoderm running down either side, for the length of the fish [15], [16]. In wildtype control fish these mesodermal stripes are clearly evident at 30 hpf (Fig. 5A), while transcript levels are reduced to near background levels in the tail by 35 hpf (Fig. 5C). The stripes are evident, albeit often patchy at 30 hpf in *gata4* morphant embryos, and embryos showed enhanced relative expression levels in the pronephric duct (Fig. 5B). More strikingly, the transcript levels persist in the *gata4* morphants and strong staining, relative to the control embryos, remains in the lateral stripes and pronephric duct at 35 hpf (Fig. 5D). Thus, *sdf1a*-dependent signaling that is normally down-regulated, may be inappropriately persistent in the *gata4* morphant embryo. We compared expression levels by quantitative RT-PCR in isolated tail tissue from control and morphant embryos at 40, 48, and 72 hpf, representing the period when normal CHT development fails in the morphants (Fig. 5E). Fully consistent with reporter fish results and *in situ* hybridization data, in morphant tissue there is a significant loss of globin transcripts (consistent with a defect in primitive red blood cell maturation), and a major decrease in transcripts for hematopoietic progenitors, including *lmo2* and *cmyb*, while both *runx1* and *sdf1a* levels are significantly increased in the tails of morphant embryos.

### Gata4 Regulates the Development of the Posterior Lateral Line

The changes in *sdf1a* expression pattern suggested a possible stromal-based defect caused by *gata4* depletion, but does not ascribe a functional role. While *Sdf1* is known for its function in mammalian hematopoiesis, it is also well characterized in zebrafish as a key regulator of the lateral line, a mechano-sensory organ system [42]. When analyzing *gata4* morphant embryos around 3–4 dpf, we discovered that they fail to initiate an “avoidance” or “startle” response, for example when touched by a pipet. The morphants are not paralyzed and swim about of their own volition. Mediation of the startle response is a major function for the lateral line. The lateral line is comprised of neuromasts, sensory organs that contain central rosettes of hair cells, similar to those found in the mammalian inner ear, surrounded peripherally by supporting cells, and innervated by sensory neurons from the cranial ganglion. The neuromasts of the posterior lateral line are derived from a primordium originating from the cephalic placodes, located posterior to the otic placodes [14]. At around 22 hpf, a primordium on each side of the embryo, consisting of ∼100 cells, migrates distally along the lateral body wall, reaching the tip of the tail by 48 hpf. During the migration, groups of cells from the trailing edge of the primordium are deposited, every 5–6 somites, on each side of the embryo. These cells, the proneuromasts, differentiate into either hair cells or supporting cells. The neuromasts integrate mechano-sensory signals that control balance and the startle response. Therefore, the *gata4* morphants display a behavior expected for embryos with a defective lateral line.

**Figure 6:**
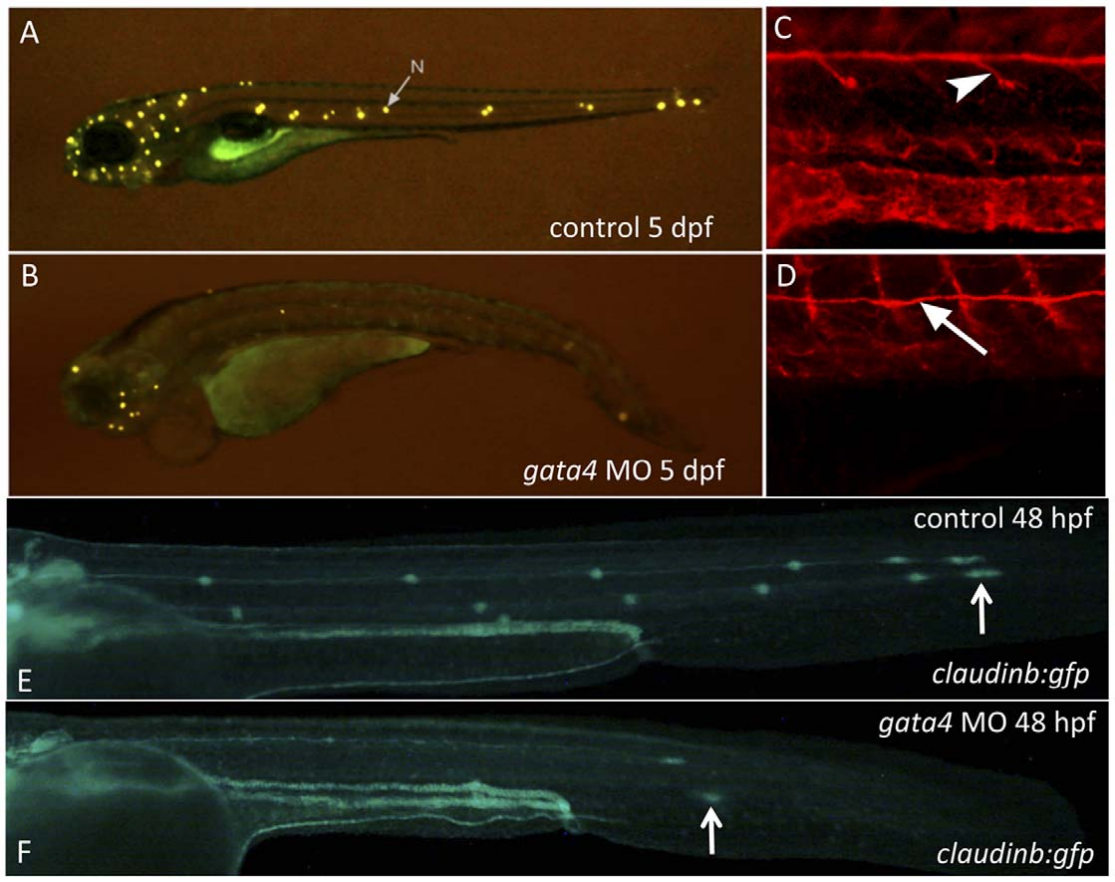
The *gata4* morphants fail to develop neuromasts of the lateral line due to a failure in neuromast deposition. A–D: Shown are representative control (A) and *gata4* morphant (B) embryos (n >50) that were stained at 5 dpf with 4-Di-2-Asp to detect differentiated neuromasts (the arrow in panel A points to a representative neuromast, N). Control (C) or morphant (D) embryos were stained with an anti-acetylated tubulin antibody to detect the neurons that migrate along with the primordium and innervate the neuromasts (arrowhead in C). While neurons fail to branch off in the morphants, the neuron tracks along the route of the primordium normally (arrow points to this neuron track in D). E–F: Shown are representative control (upper panel) and *gata4* morphant (lower panel) embryos at 48 hpf (n >50) derived from the transgenic *claudinb:gfp* reporter line, which labels the primordium and the deposited neuromasts. Specific patches of deposited cells are present at bilateral positions along the lateral line in control embryos (white arrow in E indicates the leading edge of the migrating primordium on the left side of a control embryo). In morphant embryos the primordium always migrates, and often along the normal route of the lateral line. However, in morphant embryos the migration is delayed (the arrow in F indicates the “lagging” leading edge in a representative morphant embryo), and in some morphant embryos the route was abnormal (not shown). Views are lateral, anterior to the left.

To assess the integrity of the lateral line, we first examined the development of the neuromasts. Control or morphant larvae were stained at 5 dpf in 4-Di-2-ASP, a cationic vital fluorophore that enters hair cells of the neuromasts through mechanoelectrical-transduction channels (Fig. 6A,B). The *gata4* morphant embryos display a very low number of tail neuromasts (Fig. 6B). Although variable, the anterior lateral line is less affected, and neuromasts are often found in the head regions. However, most morphant embryos lack all or most of the trunk neuromasts that comprise the posterior lateral line, and this explains their failure to generate a startle response.

While many *gata4* morphants lack all of the 4-Di-2-ASP staining trunk neuromasts, occasional neuromasts did stain. Notably, many of the morphant larvae displayed differentiated neuromasts at the extremity of the tail. Therefore, it seemed unlikely that the relative lack of trunk neuromasts is caused by a failure of the posterior lateral line primordium to migrate. Rather, we hypothesized that the primordium migrates, but that proneuromasts fail to be deposited or to differentiate normally. The migrating primordium is followed by a neurite that will innervate the future neuromasts. We reasoned that if the primordium migrates distally, the posterior lateral line neurons should be present in the *gata4* morphant embryos. To test this hypothesis, larvae at 5 dpf were analyzed by whole-mount immunohistochemistry using an anti-acetylated tubulin antibody that detects neurons (Fig. 6C,D). Indeed, the staining confirmed the existence of the posterior lateral line neurons, extending along the body length to the tail tip. This pattern suggests that the primordium migrated normally along the lateral line path. However, there was a complete lack of normal branching of these neurons toward the presumed sites of neuromast deposition, suggesting that the defect in posterior lateral line development is due to a failure in the deposition of neuromasts by the migrating primordium. We further evaluated the migration of the primordium directly using the *tg(claudinb:gfp)* reporter fish [17]. In these embryos the primordium is imaged live, seen as a migrating patch of GFP+ cells, confirmed in both control and *gata4* morphant embryos (Fig. 6E,F). As expected, the morphants generally failed to deposit proneuromast clusters. In morphant embryos the primordium often migrated along its normal route (as shown here), but it was always significantly delayed, and in some cases the primordium wandered off its normal lateral line route. Thus, in *gata4* morphants the primordium forms and migrates, but migration rate and neuromast deposition is significantly disturbed.

### Knockdown of *sdf1a* can Rescue the Lateral Line and CHT Defects Caused by Depletion of *gata4*

**Figure 7:**
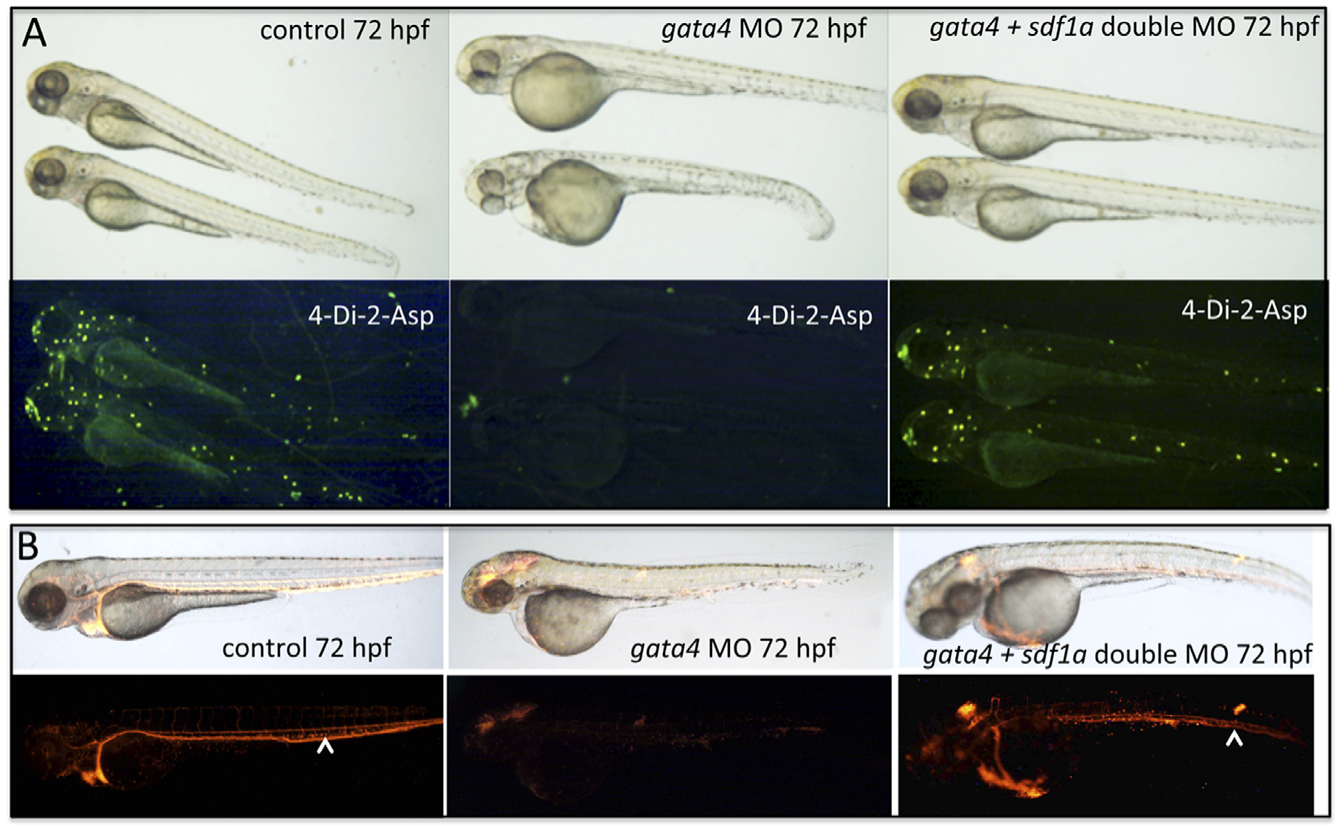
Depletion of *sdf1a* rescues the neuromast and circulation defects in the *gata4* morphant. A: Shown are representative embryos at 72 hpf (n >50, multiple independent experiments) following incubation with 4-Di-2-Asp to detect neuromasts. For comparison with control embryos (left panels) embryos were injected with the *gata4* morpholino and half the clutch was left alone (middle panels), while the other half was subsequently injected with the *sdf1a* morpholino (right panels). The lower panels are the fluorescence images of the corresponding brightfield panels. The examples shown are representative of the rescue for the lateral line in 34% of co-injected embryos (using 0.25 ng of the *sdf1a* morpholino). B: Shown are representative embryos at 72 hpf (n >50, multiple independent experiments) derived from the *tg(gata1:dsRed)* reporter line. For comparison with control embryos (left panels) embryos were injected with the *gata4* morpholino and half the clutch was left alone (middle panels), while the other half was subsequently injected with the *sdf1a* morpholino (right panels). The lower panels are the fluorescence images of the corresponding brightfield panels. The rescue of circulation into the CHT plexus in the tail was observed in 82% of co-injected embryos (using 2 ng of the *sdf1a* morpholino). White arrowheads indicate the caudal vein in the CHT. Views are lateral, anterior to the left.

The unexpected defect in lateral line development, associated here with enhanced transcript levels for *sdf1a*, prompted us to test if *sdf1a* is epistatic to *gata4*, and therefore functionally implicated in one or more of the phenotypes. If increased levels of *sdf1a*, caused by depletion of *gata4*, are functionally relevant, then reduction of *sdf1a* in the *gata4* morphant should abrogate the phenotype. In this case it is important to reduce *sdf1a* levels, but not below the threshold needed for its normal function. To test this, we used a morpholino that has been previously validated for knockdown of *sdf1a* and titrated by micro-injection to define the amount that causes reproducible lateral line defects similar to the *sdf1a* mutant [18]. We found lateral line development was normal in embryos injected with up to 4 ng of the *sdf1a* morpholino, while embryos that had been injected with 8 ng of the *sdf1a* morpholino fail to develop a lateral line. We therefore injected embryos first with the *gata4*-specific morpholino and then subsequently injected a cohort of these morphants with up to 4 ng of the morpholino targeting *sdf1a*. As little as 0.5 ng of the *sdf1a* morpholino was sufficient to effectively rescue the lateral line defect caused by loss of *gata4* (Fig. 7A), as shown by restoration of the 4-Di-2-ASP staining pattern. This amount of *sdf1a* morpholino was not sufficient to rescue CHT development (not shown). However, injecting 4 ng of *sdf1a* morpholino in the background of the *gata4* morphant was able to effectively rescue circulating blood levels, demonstrated with the *tg(gata1:dsRed)* transgenic reporter (Fig. 7B). This rescue correlates with enhanced vascularization and blood flow into the CHT. Therefore, through modulating *sdf1a* expression levels, *gata4* normally regulates lateral line and angiogenesis in the CHT. In contrast, we were unable to consistently rescue heart development in *gata4* morphants through manipulation of *sdf1a* levels (Supplemental Fig. S3).

### Gata4 Regulates *sdf1a* during Gastrulation, with Subsequent Consequences for CHT Development

While a genetic interaction between *gata4* and *sdf1a* was shown by the rescue experiments, the result is enigmatic, since neither *gata4* nor *sdf1a* is obviously expressed in the CHT. While *sdf1a* transcripts persist in the morphant tail during CHT development, *gata4* does not appear to be expressed in the tail. Although we cannot rule out low levels of expression, *gata4* and *sdf1a* are co-expressed at a much earlier stage of development, in lateral mesoderm [43], [44], [45]. To determine when *gata4* function is required for CHT development, we needed to generate a new animal model to control *gata4* activity in a conditional manner, since morpholinos knockdown *gata4* function throughout embryogenesis. For this purpose we generated a dominant-negative isoform of *gata4* and created transgenic lines of fish that could be induced to express it at any time of embryogenesis by heat shock. Our strategy was the same as one we used previously in published studies to block the function of *gata4* during *Xenopus* development [26]. Sequences encoding the well-defined DNA-binding domain of zebrafish Gata4 (amino acids 131–338) were fused with a strong-repressor domain derived from the amino terminal (amino acids 1–69) murine MXI1 transcriptional repressor protein, or a point mutant version of SR, SR-P (Supplemental Fig. S4). The SR domain of MXI1 (but not SR-P) interacts with the general chromatin repressor protein SIN3 to mediate suppression of MYC oncogenic activity [27], and when fused to a heterologous DNA-binding domain, the SR domain targets SIN3 to gene-specific binding sites leading to repression [46]. We used gel mobility-shift assays, and reporter assays to show that SR-Gata4 blocks co-expressed Gata4 from activation of target genes ([26], Supplemental Fig. S5). In preliminary experiments we also tested the homologous SR domain derived from zebrafish *mxi1*, but found no differences compared with the murine-derived domain (not shown). Injection of 250–1000 pg RNA encoding SR-Gata4 into fertilized eggs caused dose-dependent developmental defects including cardiomyopathies, whereas injection of RNA encoding SR-P-Gata4 at equivalent doses was much less active (not shown). We cloned the cDNA encoding SR-Gata4 downstream of the *hsp70I* heat-shock promoter in a tol2-dependent transgenic destination vector (Supplemental Fig. S6), and used this to generate lines of fish that express SR-Gata4, dependent on heat-shock.

**Figure 8:**
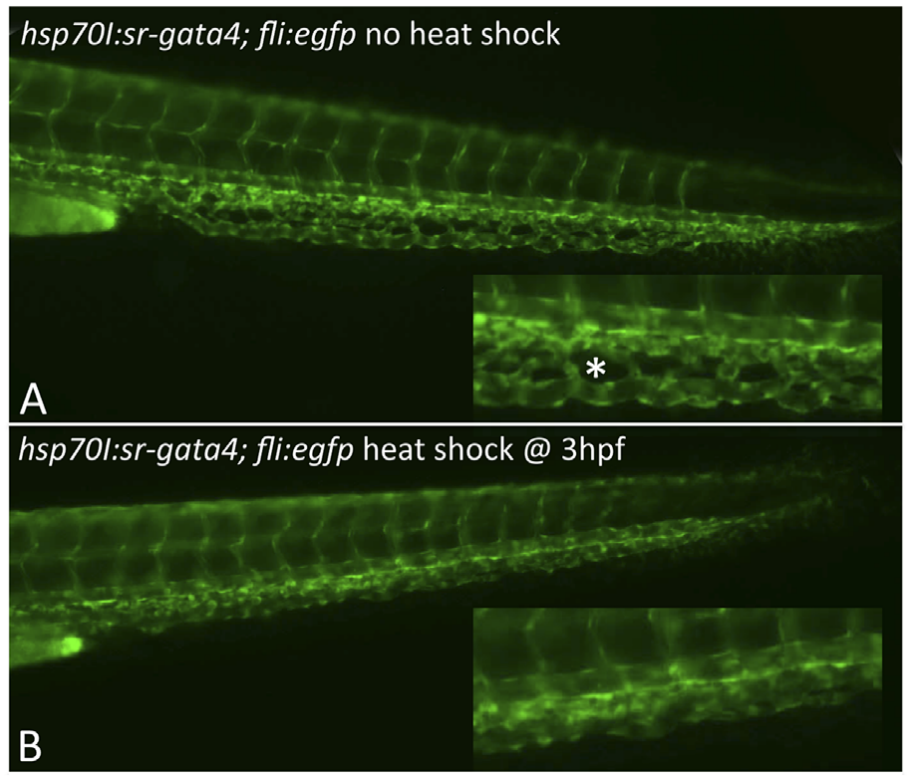
Expression of dominant-negative SR-Gata4 during gastrulation causes a block in development of the CHT plexus. Shown are representative transgenic embryos that were either left at normal temperature (A) or heat shocked for one hour at 37C starting at 3.5 hpf. Embryos are derived from crossing the *tg(sr-gata4)* line with the *tg(fli:egfp)* reporter strain. Those embryos carrying the transgene expressing SR-Gata4 are identified by RFP+ hearts (not shown here). Insets show magnified view of the equivalent regions of the developing CHT at 48 hpf. The normal fenestrated structures (an example indicated by the asterisk in A) were seen in 85% of the non-heat-shocked embryos, while in contrast, 84% of the heat-shocked embryos lacked these angiogenic structures. For each, n>50.

**Figure 9:**
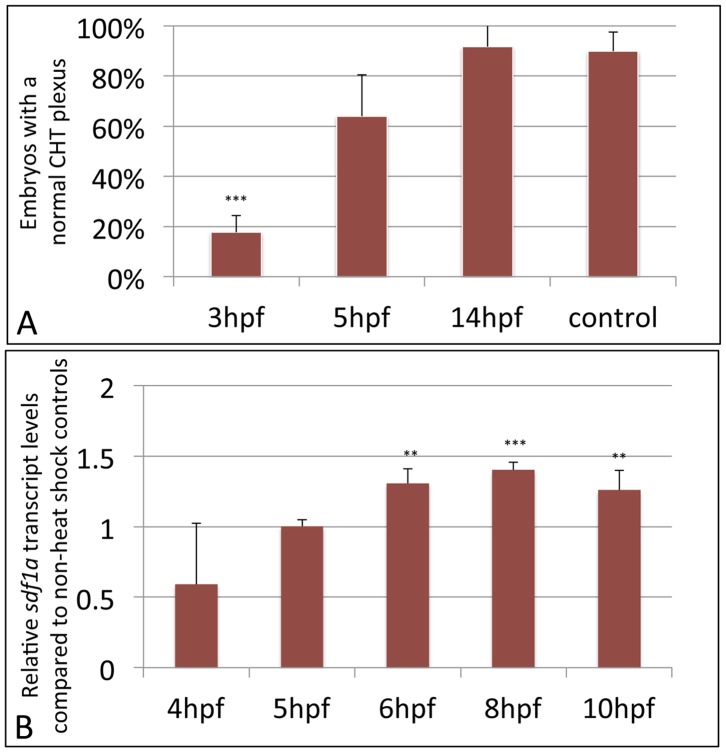
SR-Gata4 blocks CHT plexus formation and is sufficient to enhance *sdf1a* levels during epiboly, when expressed during gastrulation but not when expressed later during somitogenesis. A: The graph shows the percentage of embryos that form fenestrated structures typical of a normal CHT plexus. Embryos derived from crossing the sr-gata4 transgene into the *tg(fli:egfp)* background were either left at normal temperature (control) or heat shocked at 37C for one hour starting at 3 hpf, 5 hpf, or 14 hpf, as indicated. Because there is some variation in normal embryogenesis, the clear formation of at least 5 fenestrations, imaged at 48 hpf, was used as criteria for plexus formation. Over 80% of the embryos heat-shocked at 3 hpf failed to form a plexus (*p<0.001), while essentially all the embryos heat-shocked at 14 hpf were normal. Embryos heat-shocked at 5 hpf showed a trend toward defective plexus formation, but it was not statistically significant. For each sample, n>90, with data compiled from at least 3 independent experiments. B: Transgenic *tg(hsp70I:sr-gata4)* embryos were heat-shocked for one hour at 37C starting at 3 hpf, and batches of embryos were collected at the times indicated for analysis of *sdf1a* levels by qPCR. After normalization to beta-actin, the products were plotted relative to control non-heat shocked embryos, given an arbitrary value of 1. By 6 hpf there is a statistically significant increase in *sdf1a* transcript levels, which continues through at least 10 hpf. ** p<0.01, ***p<0.001. For each sample, n = 20–30 embryos, and the data is compiled from 4 independent experiments.

Embryos derived from the *tg(sr-gata4)* line appeared entirely normal when grown at standard 28.5C temperature. When embryos were heat-shocked for one hour at 37–39C SR-specific transcripts were readily detected by qPCR (not shown). Transgenic embryos that were heat shocked (but not sibling non-transgenic, or transgenic but non-heat-shocked embryos) developed heart and endoderm defects that phenocopy the *gata4* morphant embryos, as expected (Supplemental Fig. S6). Importantly, when heat-shocked for one hour during gastrulation, the majority of the transgenic embryos developed with a fused CHT that failed to generate a vascular plexus (Fig. 8). Heat-shock of the embryos at later stages, including 10 or 14 hpf during somitogenesis was still sufficient to generate cardiac looping defects, indicating that *gata4* function remains important during these stages for heart development. In contrast, embryos heat-shocked at the later stages are much less likely to develop defects in the CHT plexus (Fig. 9A). Importantly, heat-shock of the *tg(sr-gata4*) embryos at 3 hpf was sufficient to cause a modest but significant increase in *sdf1a* expression levels during subsequent stages of epiboly (Fig. 9B), when *sdf1a* signaling is known to control the interaction of endoderm and overlaying lateral mesoderm. Therefore, although the vascular plexus does not form until later, it appears that *gata4*-dependent regulation of *sdf1a* expression levels during gastrulation is important for mesoderm patterning relevant to CHT development.

## Discussion

In addition to its roles in cardiogenesis, gut organogenesis, and gonadal development, we discovered previously unknown functions for the transcription factor *gata4* in two additional organ systems: the vascular system and the lateral line. Recently, a functionally redundant role for *gata4/5/6* genes was shown for the development of primitive myeloid lineages [47]. However, that reflects a patterning role in lateral mesoderm for the generation of anterior primitive hemangioblasts, distinct from the non-redundant role of *gata4* we describe here. With respect to blood development, embryos depleted of *gata4* do not develop a normal vascular niche that is required during the transition from primitive to definitive lineages (the CHT), and this can explain the failure in these embryos to expand or maintain early progenitors and a lack of blood as the initial wave of primitive cells declines. The lateral line defect prompted an evaluation of *sdf1a*, which we found to be over-expressed in the *gata4* morphants. Functional epistatic tests showed that both lateral line and CHT defects can be reversed in the *gata4* morphants by depleting *sdf1a* levels, thus confirming a common, presumably non-cell-autonomous, signaling defect. In contrast, the cardiac and gut phenotypes, which may require *gata4* cell-autonomously, are not rescued by reduction of *sdf1a* levels.

In lateral line development the mechanism of *sdf1a* function has been studied extensively [48]. Active *sdf1a*-dependent *cxcr4* signaling is required at the leading edge for directed migration of the entire primordium, while *cxcr7* interprets *sdf1a* levels distinctly at the trailing edge where the release of proneuromast clusters is facilitated. In *gata4* morphants the primordium migrates, consistent with the presence of *sdf1a*. However neuromasts fail to be deposited, which is consistent with excessive Sdf1a ligand and the inability of the *cxcr7* receptor to interpret the gradient needed for deposition. The *sdf1a*-dependent signaling impacts the relative adhesion of interacting tissues. During gastrulation this controls the extent of anterior endoderm migration, relative to associated mesoderm [44], [45]. This serves to “tether” the tissues in an appropriate position, at least in part through the stimulated expression of integrins, thus facilitating spatial control of subsequent inductive signals across germ layers. While the previous work documented that disruptions in this signaling-dependent germ layer interaction alters endoderm patterning and subsequent endoderm organogenesis, our data indicate that it also affects mesoderm patterning and subsequent vascular development of the CHT.

While gastrulation and lateral line development involve collective cell migrations, it was possible that the defect in hematopoiesis could be due to a cellular chemo-attraction issue, reflecting a function more similar to that described in bone-marrow homing of hematopoietic stem cells [49]. Indeed, the expression and function of *sdf1a* in the adult zebrafish kidney is well conserved with the mammalian bone marrow program [50]. The *cxcr4a* gene facilitates regional patterning of the early aortic endothelium [51], although in this case mediated by *sdf1b*. Inappropriate levels of Sdf1a ligand in the region of the pronephros could in principle disrupt the normal migration pattern of definitive progenitors to the CHT. However, our data suggest that the defect can be explained instead by a mesoderm patterning defect leading to morphological disruption in the CHT vasculature. By 36 hpf we found in *gata4* morphants aberrant expression of *spp1* and *runx1* in the CHT, again suggesting that normal mesoderm patterning was disrupted by loss of *gata4* function. This tissue fails to be fully vascularized by 3 dpf, and so hematopoietic progenitors would be unable to seed the tissue regardless of misplaced signaling cues. To gain insight into when *gata4* expression is required to regulate CHT development we needed to develop a strategy for conditional depletion of *gata4*. This was accomplished through expression of a dominant-negative isoform of Gata4 (SR-Gata4) under the control of the heat shock promoter. The CHT failed to form a normal vascular plexus when *gata4* function was disturbed during gastrulation, while it formed normally if SR-Gata4 was expressed later during somitogenesis. Cardiac development was disrupted when SR-Gata4 is expressed at either time point. Since the SR (repressor) isoform phenocopied loss of *gata4*, including enhanced levels of *sdf1a*, the result is consistent with a normal function for *gata4* to activate gene(s) that restrict normal *sdf1a* expression levels (indirectly). Interestingly, a recent cell-tracking analysis in the zebrafish *cmyb* mutant documented defects in migration of the HSC from the dorsal aorta, which was attributed at least partly to elevated expression levels of *sdf1a* [52]. This fits well with the HSC phenotype observed in the *gata4* morphants, suggesting that mesoderm patterning may not be the only cause for the failure of HSCs to migrate to the CHT.

We speculate that one role of the CHT may be functionally homologous to the mammalian liver, as a transition zone to support hematopoiesis prior to establishment of the adult stem cell populations in the bone (mammals) or kidney (fish). The same gene, *gata4*, is an essential regulator of liver growth and development in both mammals and fish, and therefore may represent an ancient link to the evolution of hematopoietic niches. Whether the *gata4*-*sdf1a* axis is conserved in relation to murine fetal hematopoiesis is not known, although Palis and colleagues [53] showed that hematopoietic progenitors in the fetal liver are responsive to an SDF1 gradient. Furthermore, in separate studies we used a murine embryonic stem cell model that allows conditional control of *Gata4* expression [12]. When murine *Gata4* was expressed for 6 hours starting at day 2 in embryoid body (EB) cultures, the top most down-regulated gene was *Sdf1*, based on microarray analyses, and confirmed by qPCR (not shown). Thus, it seems likely that *Gata4* has a conserved role for restricting *Sdf1* during embryonic development.

## Supporting Information

Figure S1 **The CHT defect caused by depletion of Gata4 is not seen in other models of embryonic cardiomyopathy.** The heart and caudal tail regions are shown in representative 48 hpf *tg(gata1:dsred; fli1:egfp)* uninjected control embryo (top), or embryos that had been injected with morpholinos targeting *gata5* (middle) or *tbx2a* (bottom). In the trunk images, white arrows indicate examples of characteristic fenestrated structures that start to form around 32 hpf in the CHT plexus, but fail to do so in the *gata4* morphant. The control embryo has a normally looped heart, while the *gata5* morphant has an unlooped heart-string (indicated by the double-headed arrow), and the *tbx2a* morphant has an extended dysmorphic atrium (indicated by the yellow arrow). Morpholinos were used under conditions that generate reproducible cardiomyopathies and poor circulation with pericardial edema, as documented in our own and others studies: *gata5*, 5′-AAGATAAAGCCAGGCTCGAATACAT, 10 ng/embryo; *tbx2a*, 5′-CGGTGCATCCAACAAACGTAGTGAA, 5 ng/embryo.
(TIF)

Figure S2 **Progenitors that develop in association with the dorsal aorta fail to populate the CHT.** Shown are representative embryos (n >50) derived from *tg(cd41:gfp)* transgenic reporter fish, in which GFP expression marks the wave of definitive progenitors that are born in the floor of the dorsal aorta (indicated by the arrows in A, B). At least some of these migrate and seed the CHT of control embryos (boxed area in A). While the appearance of GFP+ cells from the dorsal aorta appears normal in the *gata4* morphant embryos (arrows in B), GFP+ cells are never found in the morphant CHT (equivalent boxed area in B). A’ and B’ are enlarged views of the dashed boxes shown in A and B. Views are lateral, anterior to the left.
(TIF)

Figure S3 **Modulation of Sdf1a is not sufficient to rescue the cardiac defect of** ***gata4*** **morphants.** A) Control uninjected embryos at 2 dpf have normal looped hearts, as visualized in the *tg(myl7:gfp)* transgenic reporter background. B) Hearts fail to loop in the *gata4* morphant embryos. C) Likewise, the hearts fail to loop properly in *gata4* morphants that are co-injected with the *sdf1a* morpholino. In the example shown here, 2 ng of *sdf1a* morhoplino was used, but we also failed to reproducibly rescue cardiogenesis with higher or lower doses.
(TIF)

Figure S4 **Schematic illustration of the cDNA encoding a dominant-negative SR-Gata4.** Shown on top are the previously studied versions of SR-Gata4 that was used to block *Xenopus* Gata4 function (ref. 26). The SR-P domain contains a single base change causing a valine to proline missense mutation, largely eliminating the repressor activity. PCR was used to generate the analogous sequence from the zebrafish cDNA, which was sub-cloned in frame with the SR or SR-P sequences. On the right is an autoradiogram after SDS-PAGE of lysates following in vitro transcription/translation, showing the correct expression of approximately 31 kD predicted molecular weight proteins encoding the zebrafish DNA-binding domain, and the somewhat smaller Xenopus version. The control lane sample omitted RNA.
(TIF)

Figure S5 **The SR-Gata4 proteins bind with specificity to a cognate GATA-binding site element.** Shown is an autoradiograph following electrophoresis of a specific probe containing a strong GATA-binding consensus element, following incubation with lysates generated using the *in vitro* translated *Xenopus* SR-Gata4 (SR-xG4), the zebrafish SR-Gata4 (SR-zG4), or the zebrafish SR-P-Gata4 (SR-P-zG4) proteins. For each set, the first lane lacked competitor DNA. Subsequent lanes had samples with 10× or 20× molar concentration of the specific (S-comp) or mutated non-specific (NS-comp) double-stranded competitor oligomers added to the reaction. Note that 10× of the specific competitor nearly abolishes the complex binding to the probe, while 10× of the non-specific DNA has no effect. Probe indicates the migration of free probe compared to the higher DNA-protein complexes. The larger zebrafish-derived proteins show a higher shift.
(TIF)

Figure S6 **Conditional expression of SR-Gata4 phenocopies the** ***gata4*** **morphant.** A) The schematic shows the transgenic construct used to generate lines of *tg(hsp70I:sr-gata4)* zebrafish. In the context of a tol2-flanked destination vector, the *hsp70I* promoter was used to direct expression of SR-Gata4, followed by an IRES that allows co-expression of RFP (cherry). An *myl7* promoter directing expression of GFP to the heart is also present as a marker for transgenesis. B) Shown are representative examples of three cohorts of embryos obtained by crossing zebrafish heterozygous for the transgene, imaged under fluorescence with the green channel. The top embryo is GFP+, indicating that the embryo carries the transgene. This embryo was also heat-shocked at 37C for one hour, in this case at 5, 24, and 27 hpf. The embryo in the middle was treated the same, but is GFP-, indicating that this sibling does not carry the transgene. The bottom embryo is GFP+ (transgenic) but was kept at 28.5C (no heat shock, HS-). Note that only the top embryo shows the characteristic *gata4* morphant phenotype: an improperly looped heart and pericardial edema (red arrow) and a failure in the yolk stalk extension (yellow arrow). C) The same embryos were imaged under the red channel, showing that only the top embryo expresses RFP, since it is the only embryo that is both transgenic and heat shocked. Essentially every transgenic embryo shows this heat-shock dependent phenotype (n>100).
(TIF)
