# Supplementary figures and images for: Regulation of a Vascular Plexus by gata4 Is Mediated in Zebrafish through the Chemokine sdf1a
Source: PLoS One. 2012 Oct 3;7(10):e46844. doi: 10.1371/journal.pone.0046844 (PMC3463525; doi:10.1371/journal.pone.0046844)

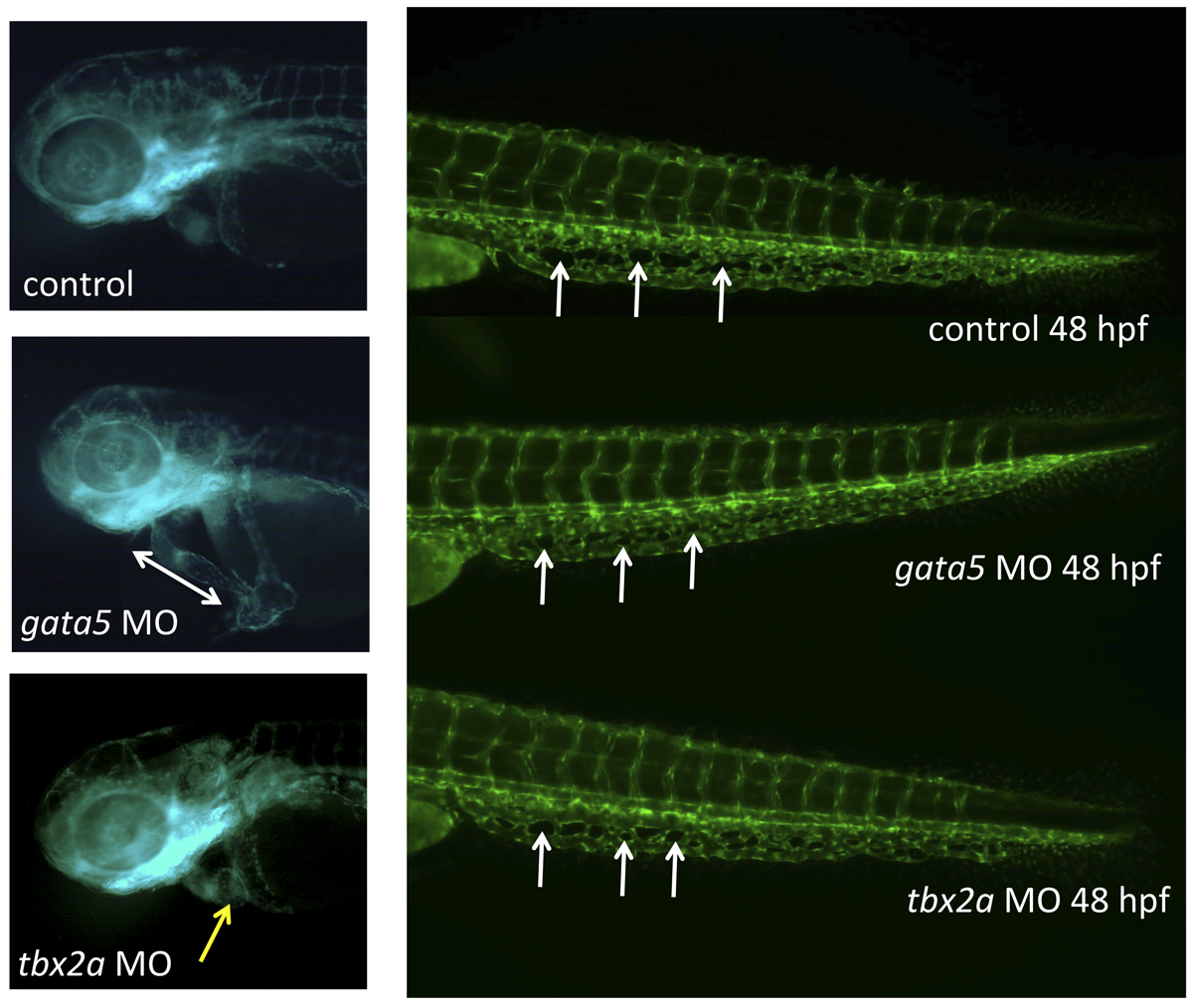

Supplement: Figure S1 — The CHT defect caused by depletion of Gata4 is not seen in other models of embryonic cardiomyopathy. The heart and caudal tail regions are shown in representative 48 hpf tg(gata1:dsred; fli1:egfp) uninjected control embryo (top), or embryos that had been injected with morpholinos targeting gata5 (middle) or tbx2a (bottom). In the trunk images, white arrows indicate examples of characteristic fenestrated structures that start to form around 32 hpf in the CHT plexus, but fail to do so in the gata4 morphant. The control embryo has a normally looped heart, while the gata5 morphant has an unlooped heart-string (indicated by the double-headed arrow), and the tbx2a morphant has an extended dysmorphic atrium (indicated by the yellow arrow). Morpholinos were used under conditions that generate reproducible cardiomyopathies and poor circulation with pericardial edema, as documented in our own and others studies: gata5, 5′-AAGATAAAGCCAGGCTCGAATACAT, 10 ng/embryo; tbx2a, 5′-CGGTGCATCCAACAAACGTAGTGAA, 5 ng/embryo. (TIF) [file pone.0046844.s001.tif]

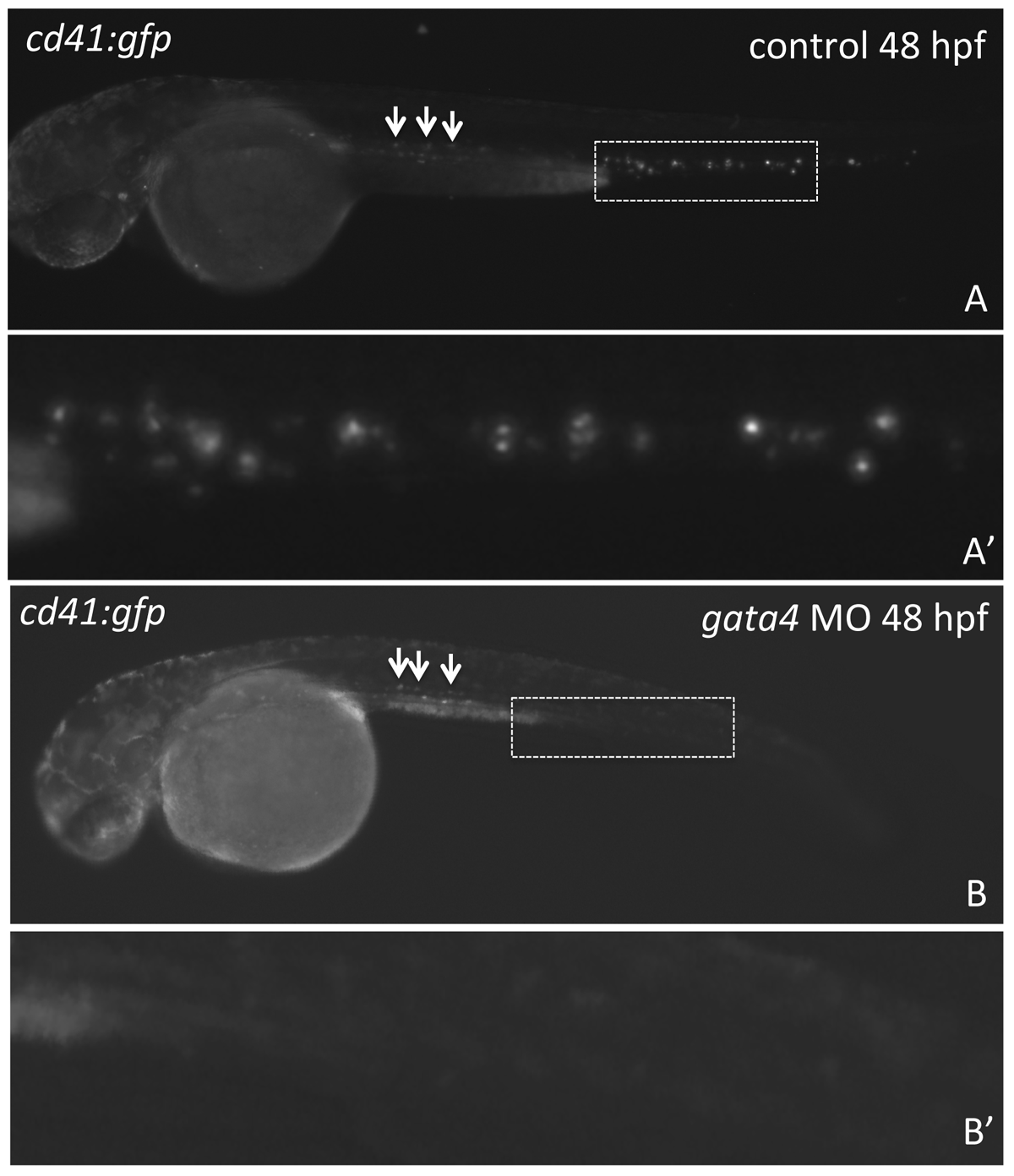

Supplement: Figure S2 — Progenitors that develop in association with the dorsal aorta fail to populate the CHT. Shown are representative embryos (n >50) derived from tg(cd41:gfp) transgenic reporter fish, in which GFP expression marks the wave of definitive progenitors that are born in the floor of the dorsal aorta (indicated by the arrows in A, B). At least some of these migrate and seed the CHT of control embryos (boxed area in A). While the appearance of GFP+ cells from the dorsal aorta appears normal in the gata4 morphant embryos (arrows in B), GFP+ cells are never found in the morphant CHT (equivalent boxed area in B). A’ and B’ are enlarged views of the dashed boxes shown in A and B. Views are lateral, anterior to the left. (TIF) [file pone.0046844.s002.tif]

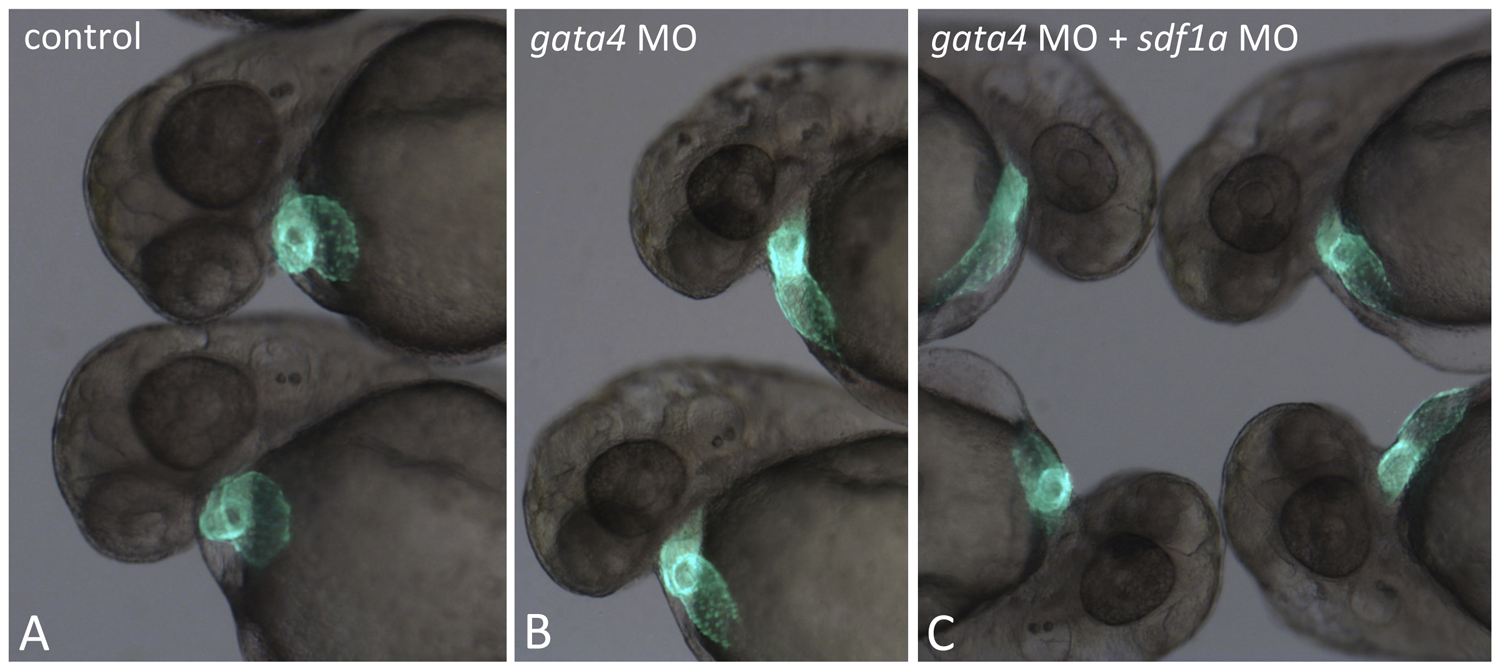

Supplement: Figure S3 — Modulation of Sdf1a is not sufficient to rescue the cardiac defect of gata4 morphants. A) Control uninjected embryos at 2 dpf have normal looped hearts, as visualized in the tg(myl7:gfp) transgenic reporter background. B) Hearts fail to loop in the gata4 morphant embryos. C) Likewise, the hearts fail to loop properly in gata4 morphants that are co-injected with the sdf1a morpholino. In the example shown here, 2 ng of sdf1a morhoplino was used, but we also failed to reproducibly rescue cardiogenesis with higher or lower doses. (TIF) [file pone.0046844.s003.tif]

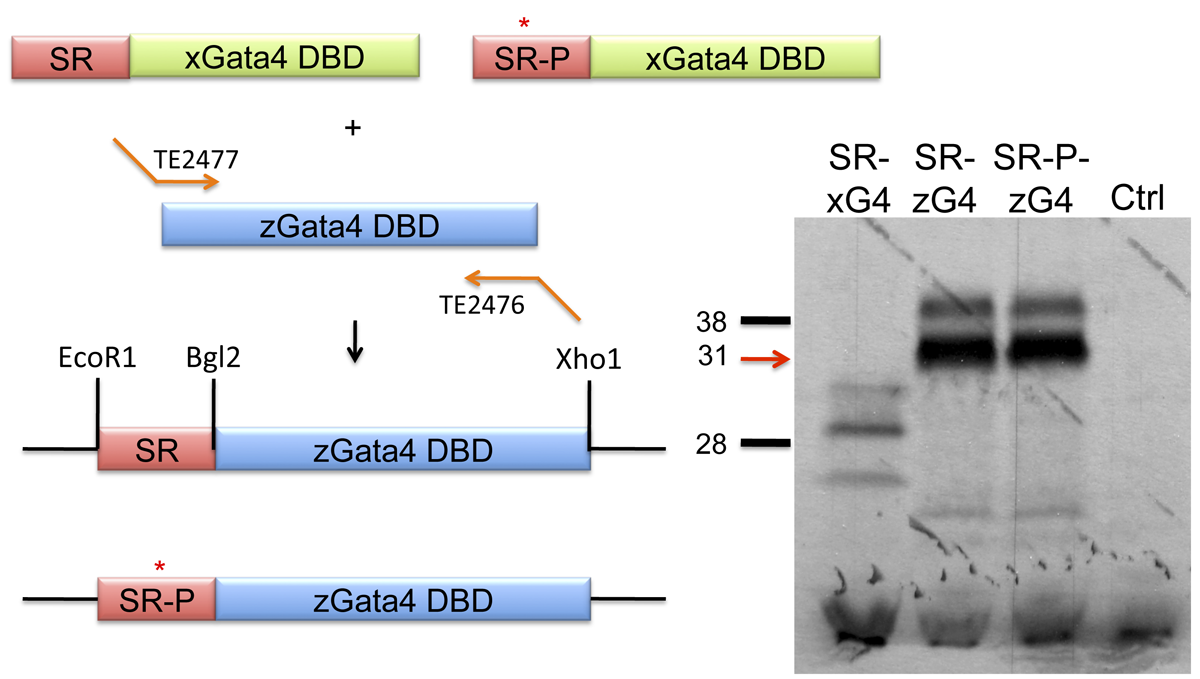

Supplement: Figure S4 — Schematic illustration of the cDNA encoding a dominant-negative SR-Gata4. Shown on top are the previously studied versions of SR-Gata4 that was used to block Xenopus Gata4 function (ref. 26). The SR-P domain contains a single base change causing a valine to proline missense mutation, largely eliminating the repressor activity. PCR was used to generate the analogous sequence from the zebrafish cDNA, which was sub-cloned in frame with the SR or SR-P sequences. On the right is an autoradiogram after SDS-PAGE of lysates following in vitro transcription/translation, showing the correct expression of approximately 31 kD predicted molecular weight proteins encoding the zebrafish DNA-binding domain, and the somewhat smaller Xenopus version. The control lane sample omitted RNA. (TIF) [file pone.0046844.s004.tif]

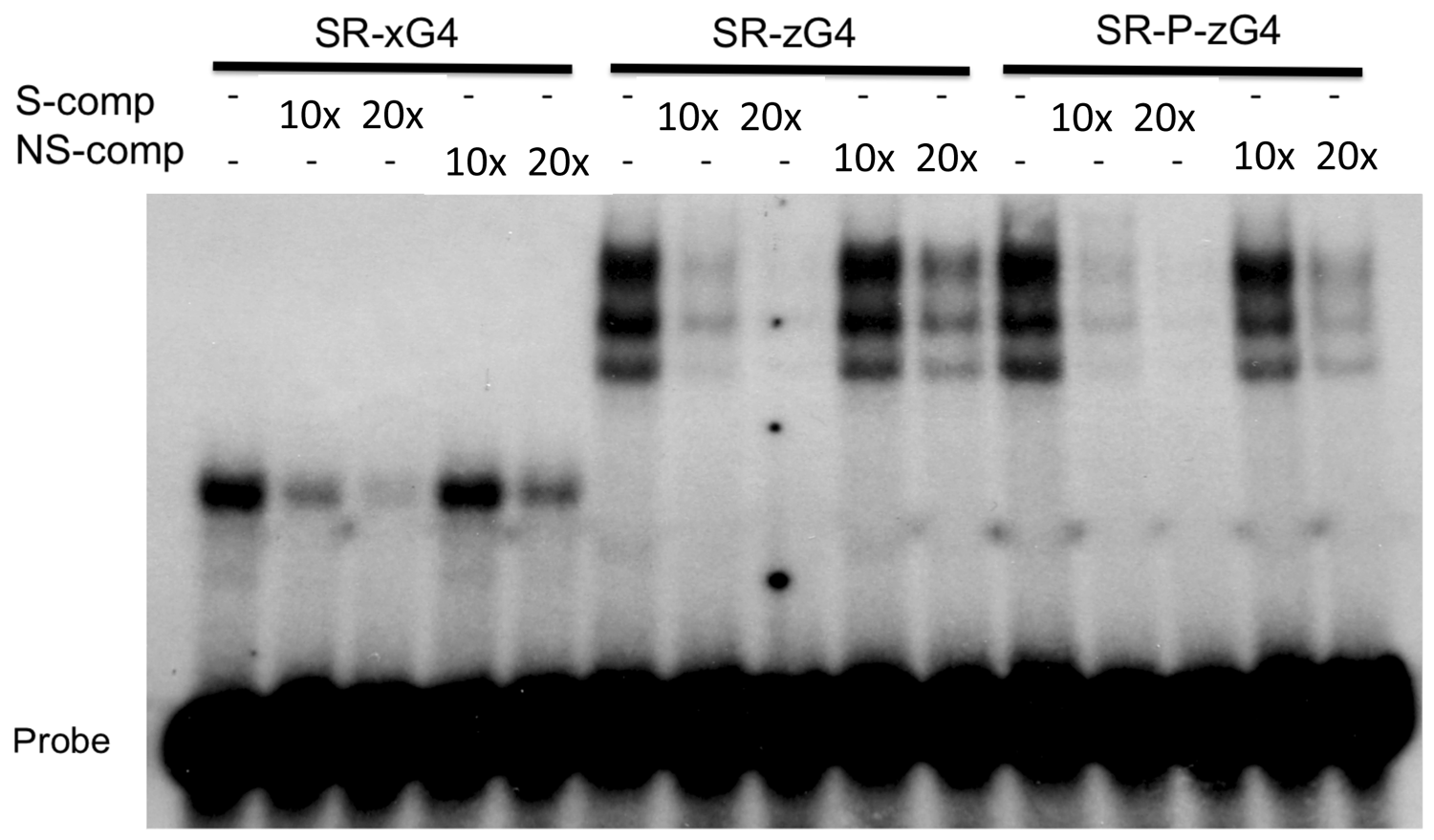

Supplement: Figure S5 — The SR-Gata4 proteins bind with specificity to a cognate GATA-binding site element. Shown is an autoradiograph following electrophoresis of a specific probe containing a strong GATA-binding consensus element, following incubation with lysates generated using the in vitro translated Xenopus SR-Gata4 (SR-xG4), the zebrafish SR-Gata4 (SR-zG4), or the zebrafish SR-P-Gata4 (SR-P-zG4) proteins. For each set, the first lane lacked competitor DNA. Subsequent lanes had samples with 10× or 20× molar concentration of the specific (S-comp) or mutated non-specific (NS-comp) double-stranded competitor oligomers added to the reaction. Note that 10× of the specific competitor nearly abolishes the complex binding to the probe, while 10× of the non-specific DNA has no effect. Probe indicates the migration of free probe compared to the higher DNA-protein complexes. The larger zebrafish-derived proteins show a higher shift. (TIF) [file pone.0046844.s005.tif]

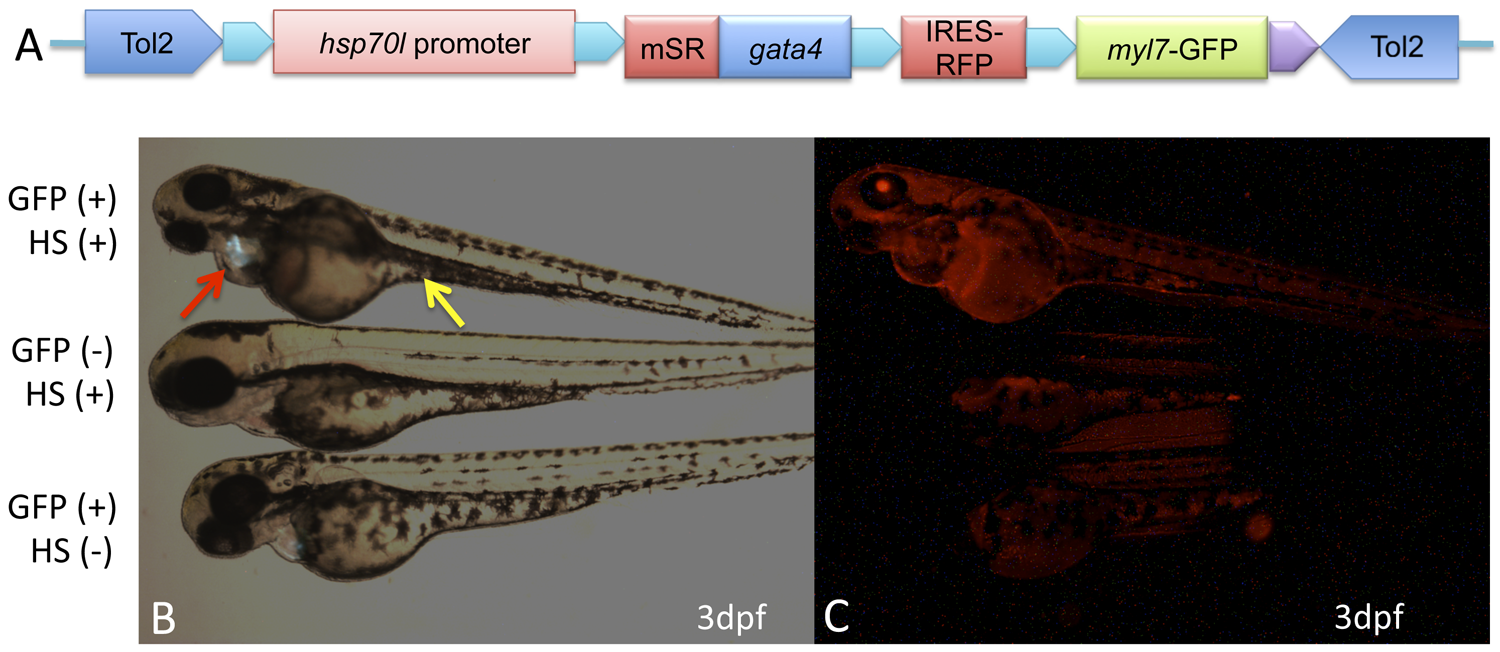

Supplement: Figure S6 — Conditional expression of SR-Gata4 phenocopies the gata4 morphant. A) The schematic shows the transgenic construct used to generate lines of tg(hsp70I:sr-gata4) zebrafish. In the context of a tol2-flanked destination vector, the hsp70I promoter was used to direct expression of SR-Gata4, followed by an IRES that allows co-expression of RFP (cherry). An myl7 promoter directing expression of GFP to the heart is also present as a marker for transgenesis. B) Shown are representative examples of three cohorts of embryos obtained by crossing zebrafish heterozygous for the transgene, imaged under fluorescence with the green channel. The top embryo is GFP+, indicating that the embryo carries the transgene. This embryo was also heat-shocked at 37C for one hour, in this case at 5, 24, and 27 hpf. The embryo in the middle was treated the same, but is GFP-, indicating that this sibling does not carry the transgene. The bottom embryo is GFP+ (transgenic) but was kept at 28.5C (no heat shock, HS-). Note that only the top embryo shows the characteristic gata4 morphant phenotype: an improperly looped heart and pericardial edema (red arrow) and a failure in the yolk stalk extension (yellow arrow). C) The same embryos were imaged under the red channel, showing that only the top embryo expresses RFP, since it is the only embryo that is both transgenic and heat shocked. Essentially every transgenic embryo shows this heat-shock dependent phenotype (n>100). (TIF) [file pone.0046844.s006.tif]
